# Supplementary figures and images for: Phenotypic and Genotypic Characterization and Antifungal Susceptibility of Sporothrix schenckii sensu stricto Isolated from a Feline Sporotrichosis Outbreak in Bangkok, Thailand
Source: J Fungi (Basel). 2023 May 18;9(5):590. doi: 10.3390/jof9050590 (PMC10219069; doi:10.3390/jof9050590)

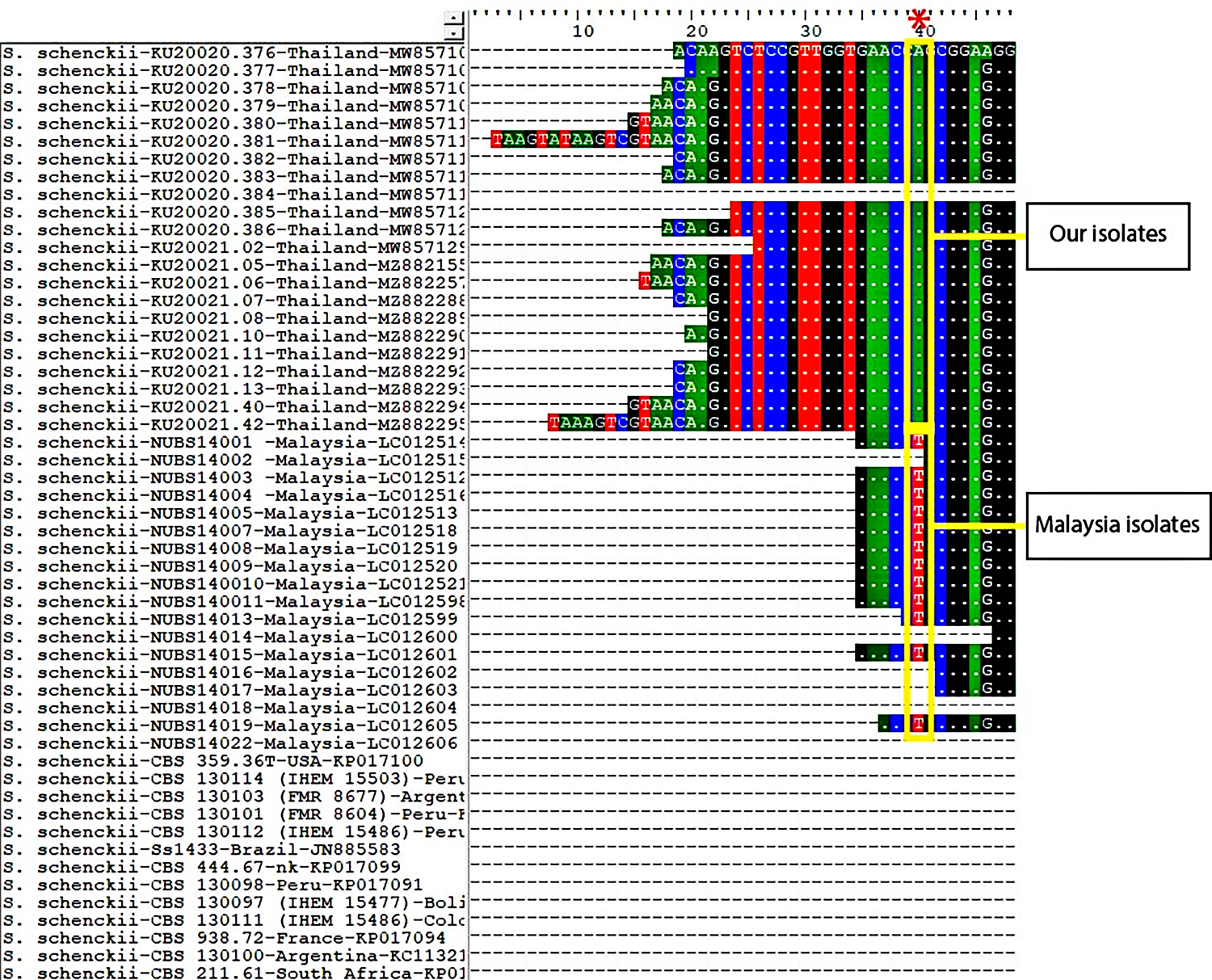

Supplement: Supplementary file 1 [file jof-09-00590-s001.zip › Figure S3.tif]

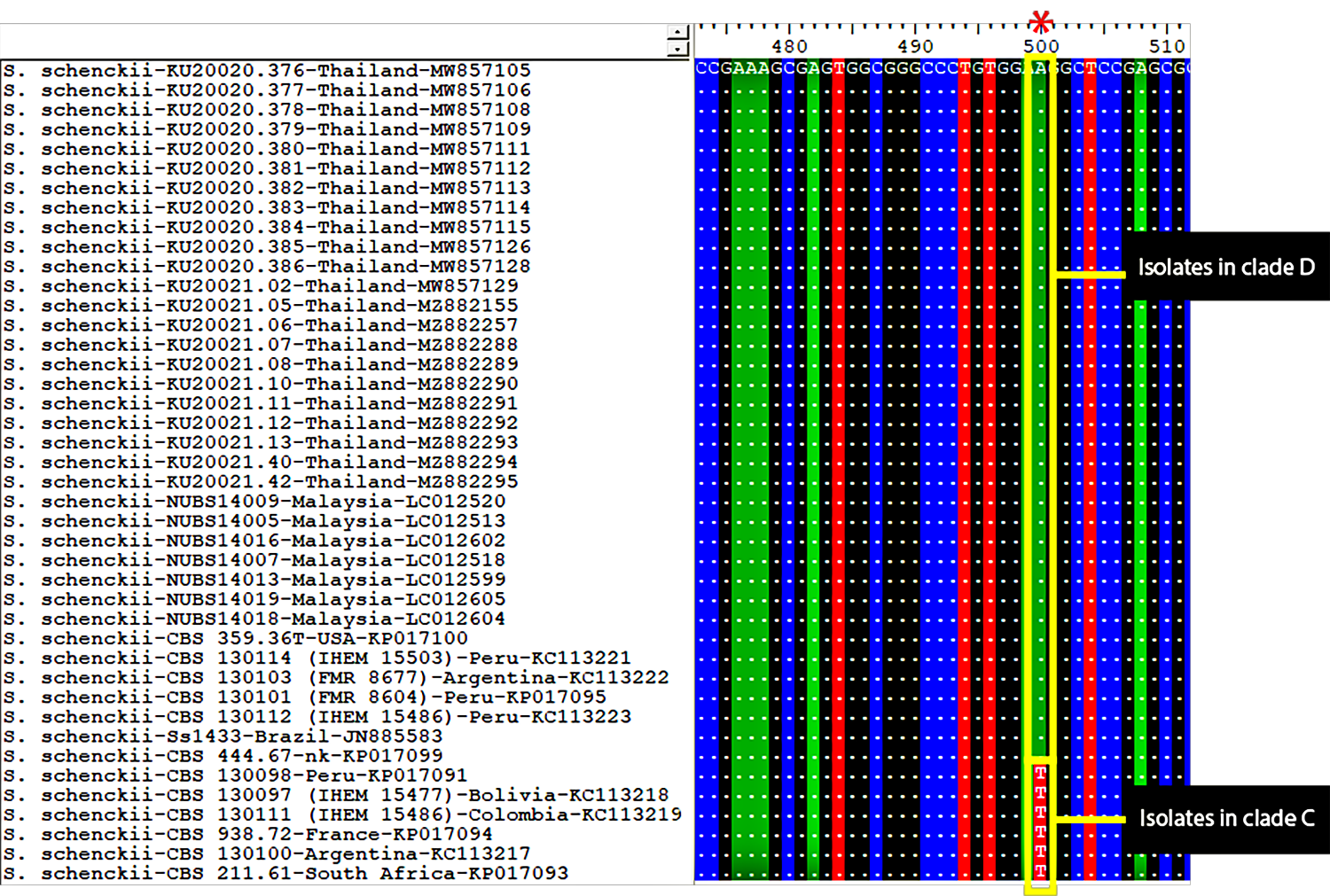

Supplement: Supplementary file 1 [file jof-09-00590-s001.zip › Figure S1.tif]

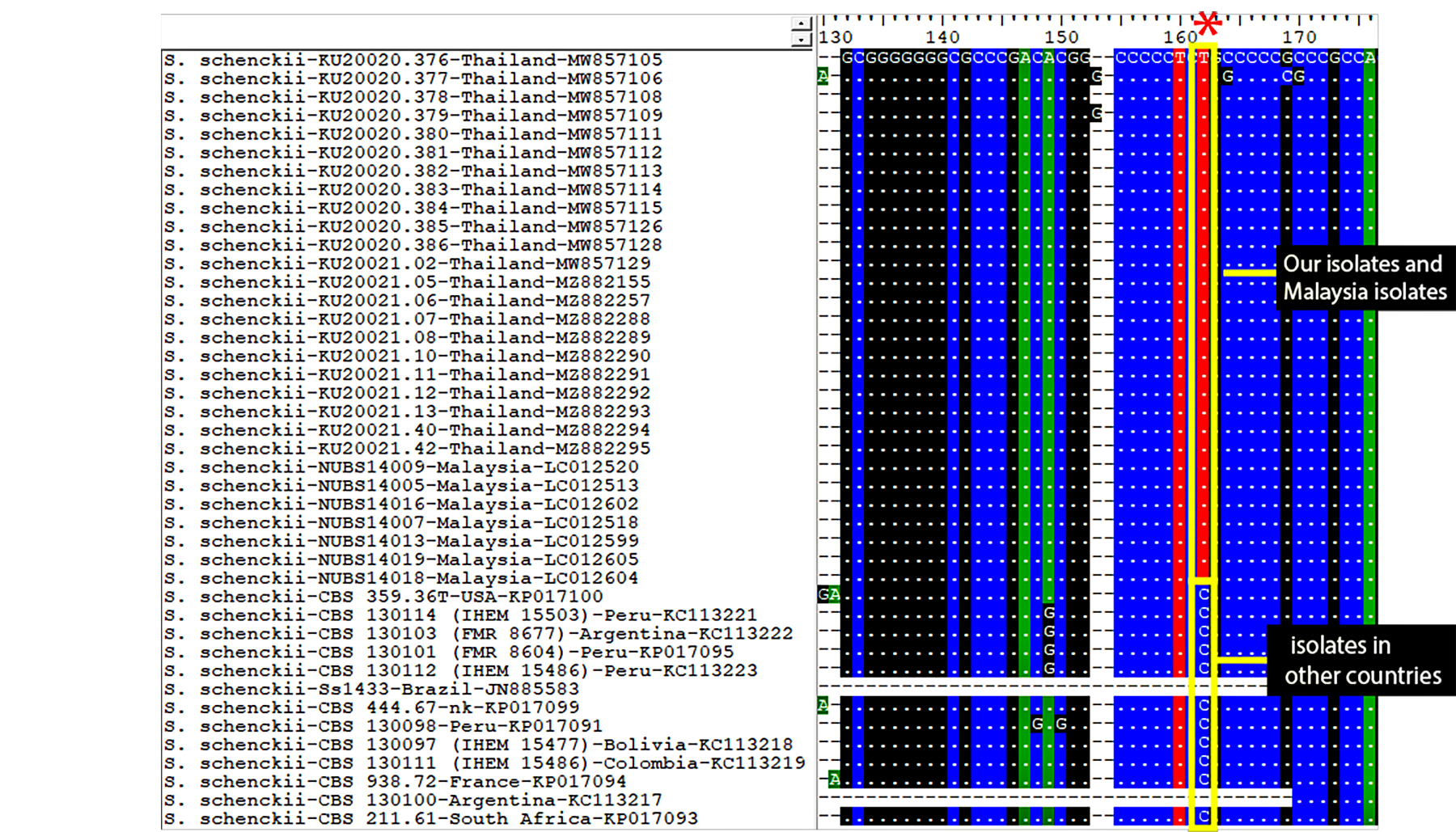

Supplement: Supplementary file 1 [file jof-09-00590-s001.zip › Figure S2.tif]
